# *Mycoplasma genitalium* antibiotic resistance-associated mutations in genital and extragenital samples from men-who-have-sex-with-men attending a STI clinic in Verona, Italy

**DOI:** 10.3389/fcimb.2023.1155451

**Published:** 2023-03-31

**Authors:** Angela Sandri, Maria Carelli, Alessandro Visentin, Alessia Savoldi, Gelinda De Grandi, Massimo Mirandola, Maria M. Lleo, Caterina Signoretto, Maddalena Cordioli

**Affiliations:** ^1^ Microbiology Section, Department of Diagnostics and Public Health, University of Verona, Verona, Italy; ^2^ School of Health Statistics and Biometrics, University of Verona, Verona, Italy; ^3^ Infectious Diseases Division, Department of Diagnostics and Public Health, University of Verona, Verona, Italy; ^4^ School of Health Sciences, University of Brighton, Brighton, United Kingdom

**Keywords:** sexually transmitted infection, *Mycoplasma genitalium*, macrolide resistance, quinolone resistance, men-who-have-sex-with-men

## Abstract

**Background:**

*Mycoplasma genitalium* (MG) is one of the most warning emerging sexually transmitted pathogens also due to its ability in developing resistance to antibiotics. MG causes different conditions ranging from asymptomatic infections to acute mucous inflammation. Resistance-guided therapy has demonstrated the best cure rates and macrolide resistance testing is recommended in many international guidelines. However, diagnostic and resistance testing can only be based on molecular methods, and the gap between genotypic resistance and microbiological clearance has not been fully evaluated yet. This study aims at finding mutations associated with MG antibiotic resistance and investigating the relationship with microbiological clearance amongst MSM.

**Methods:**

From 2017 to 2021, genital (urine) and extragenital (pharyngeal and anorectal swabs) biological specimens were provided by men-who-have-sex-with-men (MSM) attending the STI clinic of the Infectious Disease Unit at the Verona University Hospital, Verona, Italy. A total of 1040 MSM were evaluated and 107 samples from 96 subjects resulted positive for MG. Among the MG-positive samples, all those available for further analysis (n=47) were considered for detection of mutations known to be associated with macrolide and quinolone resistance. 23S rRNA, *gyrA* and *parC* genes were analyzed by Sanger sequencing and Allplex™ MG and AziR Assay (Seegene).

**Results:**

A total of 96/1040 (9.2%) subjects tested positive for MG in at least one anatomical site. MG was detected in 107 specimens: 33 urine samples, 72 rectal swabs and 2 pharyngeal swabs. Among them, 47 samples from 42 MSM were available for investigating the presence of mutations associated with macrolide and quinolone resistance: 30/47 (63.8%) showed mutations in 23S rRNA while 10/47 (21.3%) in *parC* or *gyrA* genes. All patients with positive Test of Cure (ToC) after first-line treatment with azithromycin (n=15) were infected with 23S rRNA-mutated MG strains. All patients undergoing second-line moxifloxacin treatment (n=13) resulted negative at ToC, even those carrying MG strains with mutations in *parC* gene (n=6).

**Conclusion:**

Our observations confirm that mutations in 23S rRNA gene are associated with azithromycin treatment failure and that mutations in *parC* gene alone are not always associated with phenotypic resistance to moxifloxacin. This reinforces the importance of macrolide resistance testing to guide the treatment and reduce antibiotic pressure on MG strains.

## 1 Introduction

*Mycoplasma genitalium* (MG) is among the smallest known bacteria, with a genome measuring 580 kbp, and its importance as a sexually transmitted pathogen has increased in the last decade (Blanchard and Bébéar, 2011; Fookes et al., 2017; Gnanadurai and Fifer, 2020). From a clinical perspective, MG infection can cause acute and chronic non-gonococcal urethritis (Gnanadurai and Fifer, 2020), cervicitis and the related complications such as epididymitis, pelvic inflammatory disease, preterm birth and spontaneous abortion in men and women respectively (Lis et al., 2015; Slifirski et al., 2017; Olson et al., 2021; Jensen et al., 2022). In the last years, MG has been identified as responsible pathogen for proctitis in subjects tested negative for gonococcal and chlamydial infection at anorectal site (Jensen et al., 2022). This is particularly frequent amongst men who have sex with men (MSM) population, which presents an 8-fold risk of MG infection at rectal site compared with general population (Gnanadurai and Fifer, 2020). However, MG may also be asymptomatic in both men and women (Jensen et al., 2022), and it has been estimated that up to 70% of MG infection at rectal site would be missed if not tested (Read et al., 2019; Jensen et al., 2022). According to the anatomical site, indeed, different biological samples have to be collected for MG microbiological testing: first void urine, urethral and vaginal swabs at genital site, and rectal swab at anorectal site. Oropharyngeal swab is not recommended, although it can be considered on a case-by-case basis (Jensen et al., 2022).

Given the small genome of MG and the related biosynthesis limitations, standard methods for diagnostics are not suitable in routine clinical practice (Shipitsyna et al., 2010). Indeed, MG culture may take up to two months and has low sensitivity (Hamasuna et al., 2007). For these reasons, nucleic acid amplification tests (NAATs) are nowadays the gold standard method for MG detection as the high analytical characteristics of these tests and their rapid turn-around-time improve clinical utility in real life settings (Baseman et al., 2004; Gaydos, 2017). Despite these diagnostic improvements, MG remains a clinical challenge as the treatment options are limited and hampered by the increase of antibiotic-resistant strains. Lacking the cell wall, MG is innately resistant to beta-lactams (penicillins and cephalosporins), and its ability in acquiring macrolide and quinolone resistance requires a responsible use of antibiotics (Jensen and Bradshaw, 2015).

According to the European guidelines, azithromycin represents the first-line treatment for uncomplicated MG infection in absence of macrolide resistance associated mutations or when it is not possible to perform resistance testing (Jensen et al., 2022). This antibiotic belongs to the macrolide class, that inhibits protein synthesis by binding to the A2058 and A2059 residue of 23S rRNA of the 50s ribosomal subunit (Parnham et al., 2014). Several mutations in the 23S rRNA gene (including A2058G, A2058T, A2058C, A2059G and A2059C) are known to alter the ribosome binding site of macrolides in the V region leading to resistance (Bissessor et al., 2015) and decreasing the macrolide cure rate (Jensen et al., 2022). In Europe the prevalence of azithromycin-resistant MG strains increased sharply in the last years, as recently described in a Dutch study (Hetem et al., 2021). Doxycycline, a second-generation tetracycline that acts by binding to 16S rRNA in 30S ribosomal subunit and, thus, inhibiting protein synthesis (Schnappinger and Hillen, 1996), has poor efficacy and low eradication rate (30-40%) when used in monotherapy, but it can be used as pre-treatment to reduce bacterial load and increase azithromycin success (Soni and Horner, 2019).

Treatment options for MG strains resulted positive at the macrolide resistance testing and/or those that fail microbiological clearance or clinical cure after a macrolide treatment are based on moxifloxacin use (Jensen et al., 2022). This is a second-generation fluoroquinolone that inhibits DNA gyrase (*gyrA* gene) and DNA topoisomerases IV (*parC* gene) leading to DNA disruption. Resistance to moxifloxacin is associated with mutations in the above-mentioned genes (van der Schalk et al., 2020). The overall prevalence of MG mutations associated with fluoroquinolone resistance has been reported to be 7.7%, ranging from 2.8% in the European region to 14.3% in Western Pacific regions. When both macrolide and fluoroquinolone resistance-associated mutations are considered, the overall prevalence is 2.8% (Machalek et al., 2020).

As alternative treatment options are very limited, with not optimal efficacy and low grade of evidence, a rationale use of antibiotics is warranted. Pristinamycin, a streptogramin that has a suboptimal cure rate when used in monotherapy, might have a better efficacy in sequential treatment with doxycycline (Read et al., 2018; Doyle et al., 2020). Minocycline might also be an alternative, although its efficacy in macrolide- and quinolone-resistant MG needs to be further evaluated (Soni and Horner, 2019; Doyle et al., 2020; Jensen et al., 2022).

Many studies have monitored MG antibiotic-resistance prevalence over time. A recent study involving 58 German patients reported 56.9% of samples presenting mutations associated with macrolide resistance, while 10.3% and 6.8% of detected MG carried mutations associated with fluoroquinolone and tetracycline resistance, respectively (Dumke and Spornraft-Ragaller, 2021). Amongst MSM, a French study reported antibiotic resistance-leading mutations for azithromycin and tetracycline in 67.6% and 12.5% of cases, respectively (Berçot et al., 2021). These findings highlight the importance of investigating the spread of MG antimicrobial resistance in Europe in general and in high-risk populations in particular, to improve therapeutic regimens and minimize the development and spread of antibiotic resistant strains, as recommended by international guidelines (Soni and Horner, 2019; Jensen et al., 2022). Although the gold-standard method for detection of mutations associated with antibiotic resistance is Sanger sequencing, a number of commercial tests have been developed for macrolide known resistance-associated mutations. Among them, the CE-marked *In Vitro* Diagnostic Medical Device (CE-IVD) multiplex real-time PCR Allplex MG and AziR Assay (Seegene) allows the simultaneous detection and identification of MG and 6 mutations responsible for azithromycin resistance (A2058C; A2058G; A2058T; A2059C; A2059G; A2059T). As regards fluoroquinolone resistance-associated mutations, few tests were under development and not commercially available at the time of the study (Allplex MG and MoxiR Assay, Seegene; LightMix Modular *parC* kit, TIBMOLBIOL; MGMO qPCR, NYtor).

In this study, we evaluated the presence of MG mutations associated with macrolide and fluoroquinolone resistance in biological samples collected from MSM individuals attending our STI clinic. Results may add new insights on the relationship between antibiotic resistance-associated mutations and MG microbiological clearance.

## 2 Materials and methods

### 2.1 Samples and clinical data collection

A total of 1040 MSM attending the STI clinic of the Infectious Diseases Unit of the Verona University Hospital, Verona, Italy was enrolled in the study during a 5-year period (2017–2021). Asymptomatic subjects accessing the clinic for STI screening, contacts of STI index cases, and symptomatic individuals were enrolled. The study was approved by the University Hospital of Verona Ethics Committee (1724CESC). Participants’ informed consent was obtained at enrollment. Biological samples were collected according to the purpose of the visit. For those reporting symptoms, a specimen from the site of symptoms was collected, whereas for the asymptomatic ones a gonococcal and chlamydial multi-site screening (first void urine, anorectal and pharyngeal swabs) was performed, as recommended in international guidelines (Soni and Horner, 2019; Jensen et al., 2022). Considering symptomatic MSM, gonococcal, chlamydial and MG signs and symptoms were recorded during the visit (fever, chills, dysuria, urinary urgency, pelvic pain, genital discharge, anorectal pain, tenesmus, anal discharge, inguinal lymphadenopathy).

### 2.2 Antibiotic treatment

As the study covers a 5-year period (2017-2021), in line with the international guidelines, antibiotic treatment has changed over time. According to the 2016 version of the European guidelines (Jensen et al., 2016), usually the extended azithromycin regimen (500 mg on the first day followed by 250 mg on the following 4 days) was prescribed as first-line treatment, or azithromycin in a single 1 g dose for patients with urinary infections. Moxifloxacin (400 mg orally once daily for 10 days) was used as second-line treatment for those who resulted positive at the test of cure (ToC). In 2019, considering the sequential treatment with doxycycline and macrolide or moxifloxacin recommended in the UK guidelines (Soni and Horner, 2019), these regimens were made available for treating patients. According to 2016 European guidelines, ToC was offered to all subjects 5 weeks after treatment completion.

### 2.3 DNA extraction and MG identification

Nucleic acid extraction was carried out with the fully automated Microlab Nimbus apparatus (Seegene, Inc., Seoul, Korea). The obtained extracted DNA was then tested by Anyplex II STI-7 (Seegene, Inc., Seoul, Korea) following the manufacturer’s instructions, in a CFX96 real-time thermocycler (Bio-Rad, Hercules, CA, USA). Anyplex II STI-7 (Seegene, Inc., Seoul, Korea) is an automated real-time PCR that permits the simultaneous detection of seven microorganisms that are associated, with different strength of evidence, with STIs: MG, *Chlamydia trachomatis, Neisseria gonorrhoeae, Trichomonas vaginalis, Mycoplasma hominis, Ureaplasma urealyticum, Ureaplasma parvum*. For each sample reaction, 5 μL of DNA were mixed with 15 μL of MM (master mix) and 1 μL of internal control (IC). Additionally, negative (RNase-free water) and positive controls were set up. DNA elution fractions from 47 samples resulting positive for MG were stored at −80°C and used for testing for the presence of antibiotic resistance-associated mutations by Sanger sequencing and Allplex™ MG AziR Assay (Seegene, Inc., Seoul, Korea).

### 2.4 Nested PCR and Sanger sequencing

We performed a nested PCR to partially amplify the genes of interest for detection of putative mutations associated to macrolide (region V of 23S rRNA gene) and fluoroquinolone (p*arC* and g*yrA* genes) resistance. Mutations investigated are reported in **Table S1**. The primer sequences (Pitt et al., 2018) used for nested PCR are summarized in **Table S2**. PCR reactions were performed using 5Prime Hot Master Mix (Quantabio, Beverly, MA, USA) according to manufacturer’s instructions and amplifications were carried out using AB™ Applied Biosystems GENEAMP 9700 PCR System, as shown in **Table S3** (Pitt et al., 2018). Obtained amplicons were verified by electrophoresis on 2% agarose gel followed by GelRed-staining. Amplicons were visualized by VWR™ GenoSmart 2 and sequenced by Sanger method (BMR Genomics, Padua, Italy). The resulting electropherograms were visualized and analyzed using Sequence Scanner Software v2.0 software. All samples were analyzed by Sanger sequencing.

### 2.5 AllplexTM MG AziR assay

Allplex™ MG AziR Assay (Seegene, Inc., Seoul, Korea) is a CE-IVD-marked multiplex real-time PCR capable to detect mutations in the region V of 23S rRNA gene associated to azithromycin resistance (**Table S1**) by employing differentially labeled probes. This assay was performed according to the manufacturer’s instructions using the Bio-Rad real-time CFX96™ PCR cycler (Bio-Rad Laboratories, CA, USA). Briefly, 5 μl of DNA were mixed with 15 μL of MM and 1 μL of IC. Negative (RNase-free water) and positive controls were set up. Assay run is determined as valid when the fluorophores of the positive control are ≤45 Ct value. Data were analyzed using Seegene viewer software version 3 (Seegene, Inc., Seoul, Korea). Twenty-five samples were analyzed with Allplex™ MG AziR Assay and results were compared with Sanger sequencing.

### 2.6 Statistical analysis

Mean, median, standard deviation were used for population description. For nominal variables, percentages were estimated, while Fisher’s exact test was used to estimate the association between MG detection and site. STATA Version 17 was used for analyses (College Station, TX, USA: StataCorp LP).

## 3 Results

1040 MSM attended the STI clinic of the Infectious Diseases Unit of the Verona University Hospital, Verona, Italy during the study period. 96 MSM (9.2%) resulted positive for MG in at least one anatomical site and were enrolled in line with the purpose of the study. Mean age was 37.5 ± 10.2 years, ranging from min 21 to max 57 years old. Demographic and clinical characteristics of study participants are summarized in **Table S4**.

### 3.1 MG infection and clinical outcome

The 96 study participants accounted for 107 MG-positive samples: 33/107 (33.8%) urine samples from 32/96 patients (33.3%), 72/107 (67.3%) anorectal swabs from 64/96 patients (66.7%), and 2/107 (1.9%) pharyngeal swabs from 2/96 patients (2.1%). Comparing the different sample types, MG resulted more likely to be detected at the rectal site (p<0.01). In 2 subjects, MG was detected in urine and anorectal site at the same time. Seven subjects were diagnosed with more than one MG infection across the study period (n=6 twice, n=1 thrice) and in all cases MG was detected at the same anatomical site where it was previously diagnosed. In terms of co-infections, 13/96 (13.5%) cases were observed, predominantly reflecting rectal infections (12/13): 4/96 (4.2%) patients had a concomitant *N. gonorrheae* infection, while for *C. trachomatis* the co-infection with MG was identified in 8/96 (8.3%) patients. In one case (1%), concomitant MG, gonococcal and chlamydial infection was found.

Considering signs and symptoms, 74/96 (77.1%) subjects were asymptomatic while 14/96 (14.6%) reported signs and symptoms potentially related to gonococcal, chlamydial or MG infection at genital and/or anorectal site (see **Table S4**). Considering the different sites of infection, 7/64 (10.9%) subjects with anorectal infection and 7/32 (21.9%) with urogenital infection were symptomatic. After first course of antibiotic treatment, 12/14 (85.7%) symptomatic subjects returned for ToC while, amongst those asymptomatic, only 52/74 (70.3%) returned at the clinic for having the microbiological clearance documented.

### 3.2 Antibiotic resistance-associated mutations and treatment outcome

Among the 107 MG-positive samples, 47 (n=14 urine, n=31 anorectal swabs, n=2 pharyngeal swabs) from 42 MSM were available for evaluating the presence of MG antibiotic resistance-associated mutations (reported in **Table S1**).

As regards macrolide resistance, associated mutations were detected in 30/47 (63.8%) samples. Within our samples, we detected 3 different mutations (among the 6 known) in the domain V of the 23S rRNA gene (A2059G, A2058T, A2058G). The most frequent mutation was A2059G (n=21), followed by A2058T (n=7) and A2058G (n=4); in 2 samples, both A2059G and A2058G were detected. Allplex™ MG AziR Assay and Sanger sequencing results were concordant; only in one sample, the commercial assay didn’t detect a mutation (A2059G) that was identified only through sequencing.

Mutations associated with fluoroquinolone resistance were found in 10/47 (21.3%) samples, mainly in *parC* gene (n=8). Among the latter, 6 presented SNPs/aminoacid changes of clinical relevance well known to be linked with treatment failure (**Table S1**), specifically G248T/S83I, G248A/S83N, G259A/D87N, G259T/D87Y, while two carried one not clinically relevant SNP/aminoacid change (G244A/D82N). As far as g*yrA* gene is concerned, two SNPs were identified: G285C and C270T; only the first results in an aminoacid change (M95I) and has known clinical relevance. Of note, in 8 samples among the 47 analyzed (17.0%), dual class (macrolide and quinolone) resistance-associated mutations were detected (n=6 in 23S and *parC* genes, n=2 in 23S and *gyrA* genes). No dual mutations in *gyrA* and *parC* were identified.

After azithromycin treatment, 27/42 (64.3%) subjects returned for the ToC. In all those with positive ToC after treatment with azithromycin (n=15), a mutation associated with macrolide resistance had been detected. Among patients with negative ToC after treatment with the macrolide (n=11), in most cases (n=10) no mutations in the 23s rRNA gene were detected. Only in one case azithromycin was effective in eradicating MG despite the presence of a mutation (A2058T). All patients undergoing second-line moxifloxacin treatment (n=13) resulted negative at ToC, even those carrying MG strains with mutations in *parC* gene (n=6).

## 4 Discussion

In this study, we evaluated the presence of MG mutations associated with macrolide and quinolone resistance and the outcome of the antibiotic treatment in the MSM population attending a STI clinic in Verona, Italy. The adoption of a test that simultaneously detects seven microorganisms, including MG, *N. gonorrhoeae*, *C. trachomatis* as standard diagnostic practice at the Verona University Hospital provided the opportunity to evaluate the presence of MG at genital and extragenital sites regardless of symptoms occurrence. We further investigated the presence of antibiotic resistance-associated mutations in all available samples. To our knowledge, this is the first Italian study on MG antibiotic resistance amongst MSM. Only a previous study was performed in Bari, Italy (Angela et al., 2021), considering the general population, where MG prevalence is known to be lower than in MSM. Indeed, they reported around 1% prevalence, while we found 9.2% MG prevalence in our MSM population. This is in line with previous literature, reporting a MG prevalence usually lower in the general population (1-3.3%) compared to the MSM population (3.2-35%) (Bradley et al., 2020; Gnanadurai and Fifer, 2020; Jensen et al., 2022).

Considering the different anatomical sites, as expected according to the literature (Read et al., 2019; Jensen et al., 2022) and considering the high number of asymptomatic MSM enrolled in our study (77.1%), we detected MG most frequently at rectal site (67.3% of MG-positive samples were anorectal swabs), whilst only a third (33.3%) of positive specimens were urine. These findings corroborate the body of evidence that asymptomatic MG infections is very common amongst MSM and that up to 70% of infections would be missed if the rectal site is excluded from the testing algorithm (Reinton et al., 2013; Calas et al., 2021; Jensen et al., 2022), helping the spread of MG infection in the community. It has to be noticed that, in the recent years, international guidelines narrowed testing recommendation for MG due to the lack of evidence for long-term complications and sequelae of undiagnosed and therefore untreated infections and to the MG increasing antimicrobial resistance representing a serious threat of untreatable infections (Soni and Horner, 2019; Workowski et al., 2021; Jensen et al., 2022). Indeed, although routinary multisite testing of asymptomatic MSM may in the long run slightly reduce MG prevalence and incidence, the treatment could potentially increase the levels of macrolide resistance and therefore increase the risk of making the asymptomatic populations a potential antibiotic resistance reservoir (Ong et al., 2021). In this respect, antimicrobial resistance testing should be made widely available to guide clinicians in prescribing the proper treatment and limit the spread of MG antibiotic resistance.

Conversely and in accordance with literature, the lowest MG prevalence in our study was found at pharyngeal site, with only 2.1% of MG-positive patients having a pharyngeal infection. Similarly, Latimer and colleagues (2020) found a 2% prevalence of pharyngeal infections in MSM attending the Melbourne Sexual Health Centre in Australia (Latimer et al., 2020). Although it is still debated whether pharyngeal MG is the result of passive rather than active infection, our data seems to confirm that the pharynx is not a significant infection site, as supported by the fact that pharyngeal detection is not concurrent with other sites.

In this study, we analysed macrolide and quinolone resistance-associated mutations in the available samples in order to evaluate their relationship with treatment outcome. Mutations associated with macrolide resistance, which highly correlates with treatment failure, were detected in two thirds of the considered samples (30/47, 63.8%). This finding is consistent with previous reports: globally, macrolide resistance ranges from 44% to 90% (Workowski et al., 2021) and recently, a large meta-analysis documented a sharp increase in less than a decade in macrolide resistance, from 10% before 2010 to 51% in 2016–2017 (Machalek et al., 2020). In the European region, MG macrolide resistance ranges from 3.7% to 74.3%, although Italy is not represented in this estimate (Machalek et al., 2020). To the best of our knowledge, only one study was performed in Italy, reporting a lower prevalence of azithromycin resistance-associated mutations (10.63%) compared to our (Angela et al., 2021). It has to be noted, however, that even the MG prevalence was much lower in their study (1%) than in ours (9.2%). The reason for these discrepancies might lay, on one hand, on the different population (general population *vs* MSM) and, on the other, on the different setting (general hospital *vs* STI clinic).

As far as the quinolone resistance is concerned, we detected clinically relevant mutations (Murray et al., 2022) in *parC* and *gyrA* genes in 21.3% of the analysed samples. This value is higher than the overall 7.7% prevalence reported recently reported (Machalek et al., 2020). However, there is high variability among different populations and clinical settings worldwide. Focusing on the MSM population, our data are in line with the prevalence reported in Spain (13.2%) (Fernández-Huerta et al., 2019) and in Australia (13%) (Sweeney et al., 2022), although higher values (68.3%) are reported in Japan (Ando et al., 2021). Similarly, the prevalence of dual-class-resistance-associated mutations found in our study (17%) is higher than the one reported in the systematic review (2.8%) (Machalek et al., 2020), but similar to the prevalence found in STI clinic attendees in New Zealand (20%) (Vesty et al., 2020) and in the MSM population in Australia (13-16.4%) (Murray et al., 2020; Chua et al., 2021).

As expected, in our study all azithromycin microbiological failures were observed in subjects infected with MG strains carrying macrolide-resistance associated mutations, that might have led to high-level azithromycin resistance as described in already published *in vitro* and clinical studies (Machalek et al., 2020). A possible explanation of the high prevalence of macrolide-resistant MG among MSM might be their frequent exposure to macrolide treatment due to the high burden of bacterial STI (e.g., *C. trachomatis*). In our study, 12.2% of MG-positive MSM were coinfected with *N. gonorrheae* (4.2%), *C. trachomatis* (8.3%), or both (1%), predominantly reflecting rectal infections. These findings are consistent with previous studies reporting coinfection rates of 6.3% with *N. gonorrheae*, 5.4% with *C. trachomatis* (Salado-Rasmussen et al., 2022) and an overall coinfection rate of 17% (Read et al., 2019).

As for quinolone resistance, in our study mutations in *parC* gene (G248T/S83I, G248A/S83N, G259A/D87N, G259T/D87Y) were not associated with treatment failure. Although they are frequently reported in association with reduced susceptibility to moxifloxacin or treatment unsuccess (Murray et al., 2020), their association with treatment failure is still debated. Indeed, other studies described successful eradication of MG carrying quinolone resistance-associated mutations (Murray et al., 2017). In this case, the absence of mutations in *gyrA* may play a role (Murray et al., 2023). E.g., Hamasuna and colleagues proposed that the level of MIC associated with G248T/S83I mutation may be modified by mutations in g*yrA* (Hamasuna et al., 2018).

Our data concur in emphasizing the importance of a surveillance system to collect epidemiological data linked to MG antibiotic resistance-associated mutations, like the UK pilot sentinel surveillance system “*Mycoplasma genitalium* Antimicrobial Resistance Surveillance (MARS)” (Fifer et al., 2021), to guide clinical treatment through prediction of failure and improve guidelines to slow the development and spread of resistance.

Our study has some limitations. First of all, it is a single centre study with limited sample size and therefore multicentric studies should be organised to validate our estimates. Second, the COVID-19 pandemic has reduced the frequency of MSM visits to the STI clinic and, importantly, our ability to collect and store biological samples for research purposes. As such, not all described samples could be analysed for the presence of antibiotic resistance-associated mutations. This limit, coupled with known technical difficulties in MG DNA amplification and sequencing (Pitt et al., 2020; Dumke and Spornraft-Ragaller, 2021; Guiraud et al., 2022), led to the analysis of only 47/107 samples. Third, the absence of samples with dual quinolone resistance-associated mutations impedes the evaluation of their impact on moxifloxacin treatment outcome. Fourth, as many of study participants were asymptomatic, we cannot link microbiological clearance to clinical cure. Nonetheless, our data are representative of a relevant portion of the MSM population attending the Verona STI clinic. This population should be targeted for MG testing and treatment, particularly due to the increased risk of MG antibiotic resistance.

In conclusion, our observations confirm that mutations in the 23S rRNA gene are associated with azithromycin treatment failure and that mutations in *parC* gene alone are not always associated with phenotypic resistance to moxifloxacin. This highlights the importance of timely testing for mutations associated with azithromycin resistance to improve antibiotic prescription and reduce antibiotic pressure on MG strains.

## Data availability statement

The original contributions presented in the study are included in the article/**Supplementary Material**, further inquiries can be directed to the corresponding author.

## Ethics statement

The studies involving human participants were reviewed and approved by 47837CESC AOUI Verona. The patients/participants provided their written informed consent to participate in this study.

## Author contributions

MLL and MCo contributed to conception and design of the study. ASav and MCo collected samples and recorded clinical data. ASan, MCa, and GG performed experimental activity. AV and MCa organized the database. ASan and MCa performed data and statistical analysis. MM, MLL, CS, and MCo supervised the study. ASan, MCa, and GG wrote the first draft of the manuscript. AV, ASav, MM, MLL, CS, and MCo reviewed the manuscript. All authors contributed to the article and approved the submitted version.

## References

[B36] Ando N. Mizushima D. Takano M. Mitobe M. Miyake H. Yokoyama K. (2021). High prevalence of circulating dual-class resistant mycoplasma genitalium in asymptomatic MSM in Tokyo, Japan. JAC Antimicrob. Resist. 3 (2), dlab091. doi: 10.1093/jacamr/dia091 34223146 PMC8242132

[B27] Angela A. Raffaele D. P. Federica R. Adriana M. Luigi S. Luigi R. (2021). Multi-year prevalence and macrolide resistance of mycoplasma genitalium in clinical samples from a southern Italian hospital. Eur. J. Clin. Microbiol. Infect. Dis. Off Publ. Eur. Soc. Clin. Microbiol. 40 (4), 893–895. doi: 10.1007/s10096-020-04068-3 33078220

[B11] Baseman J. B. Cagle M. Korte J. E. Herrera C. Rasmussen W. G. Baseman J. G.. (2004). Diagnostic assessment of mycoplasma genitalium in culture-positive women. J. Clin. Microbiol. 42 (1), 203–211. doi: 10.1128/JCM.42.1.203-211.2004 14715754 PMC321719

[B24] Berçot B. Charreau I. Rousseau C. Delaugerre C. Chidiac C. Pialoux G.. (2021). High prevalence and high rate of antibiotic resistance of mycoplasma genitalium infections in men who have sex with men: A substudy of the ANRS IPERGAY pre-exposure prophylaxis trial. Clin. Infect. Dis. Off Publ. Infect. Dis. Soc. Am. 73 (7), e2127–e2133. doi: 10.1093/cid/ciaa1832 33305785

[B15] Bissessor M. Tabrizi S. N. Twin J. Abdo H. Fairley C. K. Chen M. Y.. (2015). Macrolide resistance and azithromycin failure in a mycoplasma genitalium-infected cohort and response of azithromycin failures to alternative antibiotic regimens. Clin. Infect. Dis. Off Publ. Infect. Dis. Soc. Am. 60 (8), 1228–1236. doi: 10.1093/cid/ciu1162 25537875

[B1] Blanchard A. Bébéar C. (2011). The evolution of mycoplasma genitalium. Ann. N Y Acad. Sci. 1230, E61–E64. doi: 10.1111/j.1749-6632.2011.06418.x 22417108

[B28] Bradley I. Varma R. Knight V. Iliakis D. McNally L. Jalocon D.. (2020). Prevalence of rectal mycoplasma genitalium and macrolide resistance in men who have sex with men attending Sydney sexual health centre. Sex Health 17 (2), 114–120. doi: 10.1071/SH18221 31969248

[B30] Calas A. Zemali N. Camuset G. Jaubert J. Manaquin R. Saint-Pastou C.. (2021). Prevalence of urogenital, anal, and pharyngeal infections with chlamydia trachomatis, neisseria gonorrhoeae, and mycoplasma genitalium: A cross-sectional study in reunion island. BMC Infect. Dis. 21 (1), 95. doi: 10.1186/s12879-021-05801-9 33478403 PMC7818901

[B38] Chua T. P. Bodiyabadu K. Machalek D. A. Garland S. M. Bradshaw C. S. Plummer E. L.. (2021). Prevalence of mycoplasma genitalium fluoroquinolone-resistance markers, and dual-class-resistance markers, in asymptomatic men who have sex with men. J. Med. Microbiol. 70 (9), 001429. doi: 10.1099/jmm.0.001429 34590993 PMC8697509

[B22] Doyle M. Vodstrcil L. A. Plummer E. L. Aguirre I. Fairley C. K. Bradshaw C. S. (2020). Nonquinolone options for the treatment of mycoplasma genitalium in the era of increased resistance. Open Forum Infect. Dis. 7 (8), ofaa291. doi: 10.1093/ofid/ofaa291 32782911 PMC7408185

[B23] Dumke R. Spornraft-Ragaller P. (2021). Antibiotic resistance and genotypes of mycoplasma genitalium during a resistance-guided treatment regime in a German university hospital. Antibiot. Basel Switz. 10 (8), 962. doi: 10.3390/antibiotics10080962 PMC8389038 34439012

[B34] Fernández-Huerta M. Serra-Pladevall J. Barberá M. J. Espasa M. (2019). Mycoplasma genitalium and antibiotic resistance in spain; the need for an effective response against an emerging problem. Enfermedades Infecc. Microbiol. Clin. Engl. Ed. 37 (2), 144–145. doi: 10.1016/j.eimc.2018.04.008 29853280

[B43] Fifer H. Merrick R. Pitt R. Yung M. Allen H. Day M.. (2021). Frequency and correlates of mycoplasma genitalium antimicrobial resistance mutations and their association with treatment outcomes: Findings from a national sentinel surveillance pilot in England. Sex Transm. Dis. 48 (12), 951–954. doi: 10.1097/OLQ.0000000000001493 34108410

[B2] Fookes M. C. Hadfield J. Harris S. Parmar S. Unemo M. Jensen J. S.. (2017). Mycoplasma genitalium: whole genome sequence analysis, recombination and population structure. BMC Genomics 18 (1), 993. doi: 10.1186/s12864-017-4399-6 29281972 PMC5745988

[B12] Gaydos C. A. (2017). Mycoplasma genitalium: Accurate diagnosis is necessary for adequate treatment. J. Infect. Dis. ;216 (suppl_2), S406–S411. doi: 10.1093/infdis/jix104 28838072 PMC5853520

[B3] Gnanadurai R. Fifer H. (2020). Mycoplasma genitalium: A review. Microbiol. Read Engl. 166 (1), 21–29. doi: 10.1099/mic.0.000830 31329090

[B44] Guiraud J. Helary M. Le Roy C. Elguero E. Pereyre S. Bébéar C. (2022). Molecular typing reveals distinct mycoplasma genitalium transmission networks among a cohort of men who have sex with men and a cohort of women in France. Microorganisms 10 (8), 1587. doi: 10.3390/microorganisms10081587 36014005 PMC9413324

[B42] Hamasuna R. Le P. T. Kutsuna S. Furubayashi K. Matsumoto M. Ohmagari N.. (2018). Mutations in *ParC* and *GyrA* of moxifloxacin-resistant and susceptible mycoplasma genitalium strains. PloS One 13 (6), e0198355. doi: 10.1371/journal.pone.0198355 29883482 PMC5993279

[B10] Hamasuna R. Osada Y. Jensen J. S. (2007). Isolation of mycoplasma genitalium from first-void urine specimens by coculture with vero cells. J. Clin. Microbiol. 45 (3), 847–850. doi: 10.1128/JCM.02056-06 17251394 PMC1829085

[B16] Hetem D. J. Kuizenga Wessel S. Bruisten S. M. Braam J. F. van Rooijen M. S. Vergunst C. E.. (2021). High prevalence and resistance rates of mycoplasma genitalium among patients visiting two sexually transmitted infection clinics in the Netherlands. Int. J. STD AIDS. 32 (9), 837–844. doi: 10.1177/0956462421999287 33861668

[B13] Jensen J. S. Bradshaw C. (2015). Management of mycoplasma genitalium infections - can we hit a moving target? BMC Infect. Dis. 15, 343. doi: 10.1186/s12879-015.1041-6 26286546 PMC4545773

[B25] Jensen J. S. Cusini M. Gomberg M. Moi H. (2016). 2016 European Guideline on mycoplasma genitalium infections. J. Eur. Acad. Dermatol. Venereol. JEADV 30 (10), 1650–1656. doi: 10.1111/jdv.13849 27505296

[B6] Jensen J. S. Cusini M. Gomberg M. Moi H. Wilson J. Unemo M. (2022). 2021 European Guideline on the management of mycoplasma genitalium infections. J. Eur. Acad. Dermatol. Venereol. JEADV 36 (5), 641–650. doi: 10.1111/jdv.17972 35182080

[B33] Latimer R. L. Vodstrcil L. De Petra V. Fairley C. K. Read T. R. Williamson D.. (2020). Extragenital mycoplasma genitalium infections among men who have sex with men. Sex Transm. Infect. 96 (1), 10–18. doi: 10.1136/sextrans-2019-054058 31217322

[B4] Lis R. Rowhani-Rahbar A. Manhart L. E. (2015). Mycoplasma genitalium infection and female reproductive tract disease: a meta-analysis. Clin. Infect. Dis. Off Publ. Infect. Dis. Soc. Am. 61 (3), 418–426. doi: 10.1093/cid/civ312 25900174

[B20] Machalek D. A. Tao Y. Shilling H. Jensen J. S. Unemo M. Murray G.. (2020). Prevalence of mutations associated with resistance to macrolides and fluoroquinolones in mycoplasma genitalium: A systematic review and meta-analysis. Lancet Infect. Dis. 20 (11), 1302–1314. doi: 10.1016/S1473-3099(20)30154-7 32622378

[B39] Murray G. L. Bodiyabadu K. Danielewski J. Garland S. M. Machalek D. A. Fairley C. K.. (2020). Moxifloxacin and sitafloxacin treatment failure in mycoplasma genitalium infection: Association with *parC* mutation G248T (S83I) and concurrent *gyrA* mutations. J. Infect. Dis. 221 (6), 1017–1024. doi: 10.1093/infdis/jiz550 32031634

[B46] Murray G. L. Bodiyabadu K. Vodstrcil L. A. Machalek D. A. Danielewski J. Plummer E. L.. (2022). parC variants in mycoplasma genitalium: Trends over time and association with moxifloxacin failure. Antimicrob. Agents Chemother. 66 (5), e0027822. doi: 10.1128/aac.00278-22 35475636 PMC9112906

[B41] Murray G. L. Bradshaw C. S. Bissessor M. Danielewski J. Garland S. M. Jensen J. S.. (2017). Increasing macrolide and fluoroquinolone resistance in mycoplasma genitalium. Emerg. Infect. Dis. 23 (5), 809–812. doi: 10.3201/eid2305.161745 28418319 PMC5403035

[B47] Murray G. L. Plummer E. L. Bodiyabadu K. Vodstrcil L. A. Huaman J. L. Danielewski J. A.. (2023). gyrA mutations in mycoplasma genitalium and their contribution to moxifloxacin failure: Time for the next generation of resistance-guided therapy. Clin. Infect. Dis. ciad057. doi: 10.1093/cid/ciad057 36722416 PMC10273371

[B7] Olson E. Gupta K. van der Pol B. Galbraith J. W. Geisler W. M. (2021). Mycoplasma genitalium infection in women reporting dysuria: A pilot study and review of the literature. Int. J. STD AIDS. 32 (13), 1196–1203. doi: 10.1177/09564624211030040 34229513 PMC8858599

[B32] Ong J. J. Ruan L. Lim A. G. Bradshaw C. S. Taylor-Robinson D. Unemo M.. (2021). Impact of screening on the prevalence and incidence of mycoplasma genitalium and its macrolide resistance in men who have sex with men living in Australia: A mathematical model. E. Clin. Med. 33, 100779. doi: 10.1016/j.eclinm.2021.100779 PMC8020166 33842867

[B14] Parnham M. J. Erakovic Haber V. Giamarellos-Bourboulis E. J. Perletti G. Verleden G. M. Vos R. (2014). Azithromycin: Mechanisms of action and their relevance for clinical applications. Pharmacol. Ther. 143 (2), 225–245. doi: 10.1016/j.pharmthera.2014.03.003 24631273

[B26] Pitt R. Fifer H. Woodford N. Alexander S. (2018). Detection of markers predictive of macrolide and fluoroquinolone resistance in mycoplasma genitalium from patients attending sexual health services in England. Sex Transm. Infect. 94 (1), 9–13. doi: 10.1136/sextrans-2017-053164 28717051

[B45] Pitt R. Unemo M. Sonnenberg P. Alexander S. Beddows S. Cole M. J.. (2020). Antimicrobial resistance in mycoplasma genitalium sampled from the British general population. Sex Transm. Infect. 96 (6), 464–468. doi: 10.1136/sextrans-2019-054129 31924741 PMC7476295

[B21] Read T. R. H. Jensen J. S. Fairley C. K. Grant M. Danielewski J. A. Su J.. (2018). Use of pristinamycin for macrolide-resistant mycoplasma genitalium infection. Emerg. Infect. Dis. 24 (2), 328–335. doi: 10.3201/eid2402.170902 29350154 PMC5782881

[B8] Read T. R. H. Murray G. L. Danielewski J. A. Fairley C. K. Doyle M. Worthington K.. (2019). Symptoms, sites, and significance of mycoplasma genitalium in men who have sex with men. Emerg. Infect. Dis. 25 (4), 719–727. doi: 10.3201/eid2504.181258 30882306 PMC6433010

[B29] Reinton N. Moi H. Olsen A. O. Zarabyan N. Bjerner J. Tønseth T. M.. (2013). Anatomic distribution of neisseria gonorrhoeae, chlamydia trachomatis and mycoplasma genitalium infections in men who have sex with men. Sex Health 10 (3), 199–203. doi: 10.1071/SH12092 23751932

[B40] Salado-Rasmussen K. Tolstrup J. Sedeh F. B. Larsen H. K. Unemo M. Jensen J. S. (2022). Clinical importance of superior sensitivity of the aptima TMA-based assays for mycoplasma genitalium detection. J. Clin. Microbiol. 60 (4), e0236921. doi: 10.1128/jcm.02369-21 35317613 PMC9020335

[B17] Schnappinger D. Hillen W. (1996). Tetracyclines: Antibiotic action, uptake, and resistance mechanisms. Arch. Microbiol. 165 (6), 359–369. doi: 10.1007/s002030050339 8661929

[B9] Shipitsyna E. Savicheva A. Solokovskiy E. Ballard R. C. Domeika M. Unemo M.. (2010). Guidelines for the laboratory diagnosis of mycoplasma genitalium infections in East European countries. Acta Derm. Venereol. 90 (5), 461–467. doi: 10.2340/00015555-0929 20814619

[B5] Slifirski J. B. Vodstrcil L. A. Fairley C. K. Ong J. J. Chow E. P. F. Chen M. Y.. (2017). Mycoplasma genitalium infection in adults reporting sexual contact with infected partners, Australia, 2008-2016. Emerg. Infect. Dis. 23 (11), 1826–1833. doi: 10.3201/eid2311.170998 29047422 PMC5652440

[B18] Soni S. Horner P. J. (2019). Launch of the BASHH guideline for the management of M. genitalium in adults. Sex Transm. Infect. 95 (4), 237. doi: 10.1136/sextrans-2018-053831 31097546

[B35] Sweeney E. L. Bradshaw C. S. Murray G. L. Whiley D. M. (2022). Individualised treatment of mycoplasma genitalium infection-incorporation of fluoroquinolone resistance testing into clinical care. Lancet Infect. Dis. 22 (9), e267–e270. doi: 10.1016/S1473-3099(21)00629-0 35325618

[B19] van der Schalk T. E. Braam J. F. Kusters J. G. (2020). Molecular basis of antimicrobial resistance in mycoplasma genitalium. Int. J. Antimicrob. Agents. 55 (4), 105911. doi: 10.1016/j.ijantimicag.2020.105911 31991219

[B37] Vesty A. McAuliffe G. Roberts S. Henderson G. Basu I. (2020). Mycoplasma genitalium antimicrobial resistance in community and sexual health clinic patients, Auckland, new Zealand. Emerg. Infect. Dis. 26 (2), 332–335. doi: 10.3201/eid2602.190533 31961302 PMC6986824

[B31] Workowski K. A. Bachmann L. H. Chan P. A. Johnston C. M. Muzny C. A. Park I.. (2021). Sexually transmitted infections treatment guidelines, 2021. MMWR Recomm. Rep. Morb. Mortal. Wkly. Rep. Recomm. Rep. 70 (4), 1–187. doi: 10.15585/mmwr.rr7004a1 PMC8344968 34292926

