# Supplementary material for: Mycoplasma genitalium antibiotic resistance-associated mutations in genital and extragenital samples from men-who-have-sex-with-men attending a STI clinic in Verona, Italy
Source: Front Cell Infect Microbiol. 2023 Mar 31;13:1155451. doi: 10.3389/fcimb.2023.1155451 (PMC10102577; doi:10.3389/fcimb.2023.1155451)
Supplement: Supplementary file 1 [file Table_1.docx]

Supplementary Material

Mycoplasma genitalium antibiotic resistance-associated mutations in genital and extragenital samples from men-who-have-sex-with-men attending a STI Clinic in Verona, Italy

Angela Sandri, Maria Carelli, Alessandro Visentin, Alessia Savoldi, Gelinda De Grandi, Massimo Mirandola, Maria M. Lleo, Caterina Signoretto*, Maddalena Cordioli

*** Correspondence:** Caterina Signoretto, [caterina.signoretto@univr.it](mailto:caterina.signoretto@univr.it)

# Supplementary Tables

**Table S1.** Mutations associated with macrolide and quinolone resistance.

| Gene | Mutation | AA* change | Clinically relevant | Ref. |
| --- | --- | --- | --- | --- |
| 23S rRNA | A2058C |  | Yes | Jensen 2008, Twin 2012, Kaitlin 2013 |
|  | A2058G |  | Yes | Jensen 2008, Twin 2012, Kaitlin 2013, Unemo 2017, Pitt 2018 |
|  | A2058T |  | Yes | Twin 2012, Kaitlin 2013, Unemo 2017 |
|  | A2059C |  | Yes | Twin 2012, Unemo 2017, Pitt 2018 |
|  | A2059G |  | Yes | Jensen 2008, Twin 2012, Kaitlin 2013, Unemo 2017, Pitt 2018 |
|  | A2059T |  | Yes | Machalek 2020 |
| *ParC* | C234T | Silent | - | Kaitlin 2013, Gardette 2022 |
|  | G241T | G81C | Yes | Gruson 2005, Kaitlin 2013 |
|  | G244A | D82N | Na | Beeton 2009, Kaitlin 2013 |
|  | A247C | S83R | Yes | Shimada 2010, Kaitlin 2013, Pond 2014, Unemo 2017 |
|  | G248T | S83I | Yes | Gruson 2005, Kaitlin 2013, Fookes 2017, Unemo 2017, Pitt 2018 |
|  | G248A | S83N | Yes | Shimada 2010, Unemo 2017 |
|  | G259A | D87N | Yes | Shimada 2001, Gruson 2005, Kaitlin 2013, Unemo 2017 |
|  | G259C | D87H | Na | Shimada 2010, Kaitlin 2013, Unemo 2017 |
|  | G259T | D87Y | Yes | Shimada 2001, Kaitlin 2013, Pitt 2018 |
|  | A260G | D87G | Na | Gruson 2005, Kaitlin 2013 |
|  | A260T | D87V | Na | Shimada 2010 |
|  | A290G | K97R | Yes | Shimada 2011 |
|  | G307A | V103I | Na | Kaitlin 2013 |
|  | G336A | Silent | - | Dumke 2016 |
|  | T351C | Silent | - | Kaitlin 2013 |
| *GyrA* | G237T | Silent | - | Kaitlin 2013 |
|  | C270T | Silent | - | Kaitlin 2013 |
|  | G285A | Silent | - | Kaitlin 2013 |
|  | G285C | M95I | Yes | Bebear 2003, Gruson 2005, Kaitlin 2013, Hamasuna 2018 |
|  | T321A | F108I | Yes | Shimada 2010 |
|  | A288G | Silent | - | Jensen 2018 |
|  | T378C | Silent | - | Jensen 2018 |

*AA=aminoacid

**Table S2.** Primer sequences used to amplify genes of interest for sequencing.

| **Antibiotic resistance** | **Gene** | **1^st^ PCR** | | | **2^nd^ PCR** | |
| --- | --- | --- | --- | --- | --- | --- |
|  |  | **F/R*** | **Primer sequence**  **5'-3'** | **Amplicon size (bp)** | **F/R** | **Primer sequence**  **5'-3'** |
| Macrolide | 23S rRNA | F | 5'-AGT GAA CGT GTG ATC AAG TAG-3' | 785 | F | 5'- GAA GGA GGT TAG CAA TTT ATT GC -3' |
|  |  | R | 5'- T CTA AAT ACG ATT TCC AAC CG-3' |  | R | 5'- TTC TCT ACA TGG TGG TGT TTT- 3' |
| Quinolone | *GyrA* | F | 5'- GCT AGA GAT GGA CTT AAA CCA G-3' | 251 | F | 5'- TAT GGT GCT TAT ATT GGT GGC -3' |
|  |  | R | 5'- ATC TTA ATG AAA AGT AAA AGT CTT GAG CC |  | R | 5'- ATC TTA ATG AAA AGT CTT GAG CC -3' |
|  | *ParC* | F | 5'- CTT TAC CTG ATC TAA GAG ATG GG-3' | 251 | F | 5'- GCT TAA AAC CCA CCA CTC C -3' |
|  |  | R | 5'- ATT AC CCC ATC CAC TGA ACC -3' |  | R | 5'- GTT GTT CAG TTG TTC TTT CAG C -3' |

*F=Forward, R=Reverse

**Table S3.** Thermal profiles used to amplify genes of interest for sequencing.

| **PCR stage** | **23S rRNA gene** | | | | | | ***ParC* and *GyrA* gene** | | | | |
| --- | --- | --- | --- | --- | --- | --- | --- | --- | --- | --- | --- |
|  | **1^st^ PCR** | | | **2^nd^ PCR** | | | **1^st^ PCR** | | | **2^nd^ PCR** | |
|  | **Temperature (°C)** | **Time** | **Cycles** | **Temperature (°C)** | **Time** | **Cycles** | **Temperature (°C)** | **Time** | **Cycles** | **Temperature (°C)** | **Time** |
| Denaturation | 94 | 3' | 1 | 94 | 3' | 1 | 94 | 1' | 1 | 94 | 1' |
|  | 94 | 1' | 35 | 94 | 15'' | 40 | 94 | 30'' | 40 | 94 | 30'' |
| Annealing | 55 | 30'' |  | 60 | 15'' |  | 57 | 30'' |  | 57 | 30'' |
| Extension | 72 | 1' |  | 72 | 10'' |  | 72 | 1' |  | 72 | 1' |

**Table S4**. Patients and samples characteristics, including age, anatomical site, co-infections, symptoms, gene mutations identified, therapies and ToCs results

| **Patient ID** | **Age** | **Site** | **Co-infection** | **Symptoms** | **Gene mutations** | **1st therapy** | **1st ToC result** | **2nd therapy** | **2nd ToC result** |
| --- | --- | --- | --- | --- | --- | --- | --- | --- | --- |
| P1 | 57 | Anorectal swab | No | No | No | AZM ER | Neg | na | na |
| P2 | 27 | Urine | No | No | na | DOX 7d + MOX 7d | Pos | MOX 10d | Pos |
| P3 | 41 | Urine | No | na | 23S (A2058T) | na | na | na | na |
| P4 | 39 | Urine | No | Yes | 23S (A2059G) | na | Neg | na | na |
| P5 | 39 | Anorectal swab | No | No | 23S (A2059G) | MOX 10d | Neg | na | na |
| P6 | 32 | Anorectal swab | No | No | na | AZM ER | Neg | na | na |
| P7 | 29 | Anorectal swab | CT | No | 23S (A2058T) | AZM ER | Pos | MOX 10d | Neg |
| P8 | 38 | Urine | No | No | 23S (A2058G, A2059G) | AZM 1g | na | na | na |
| P9 | 25 | Anorectal swab | No | No | na | AZM ER | na | na | na |
| P10 | 26 | Urine | No | No | na | AZM 1g | Pos | MOX 10d | Neg |
| P11 | 23 | Urine | No | No | na | AZM ER | Neg | na | na |
| P12 ^1^ | 28 | Urine | No | Yes | No | AZM ER | Neg | na | na |
| P12 ^1^ | 28 | Anorectal swab | No |  | No |  | Neg |  | na |
| P12 ^2^ | 29 | Urine | No | No | 23S (A2058G) | AZM ER | Pos | AZM 1g | Neg |
| P12 ^2^ | 29 | Anorectal swab | No |  | No |  | na |  | Neg |
| P13 | 34 | Pharyngeal swab | NG | No | No | AZM ER | Neg | na | na |
| P14 | 43 | Urine | No | na | na | na | na | na | na |
| P15 | 40 | Anorectal swab | No | No | na | AZM ER | Pos | na | Neg |
| P16 | 52 | Anorectal swab | No | No | na | AZM ER | Neg | na | na |
| P17 | 45 | Anorectal swab | No | No | 23S (A2059G), *parC* (G244A) | AZM ER | Pos | MOX 10d | Neg |
| P18 | 21 | Anorectal swab | No | No | 23S (A2059G), *parC* (G248T) | AZM ER | Pos | MOX 10d | Neg |
| P19 | 46 | Anorectal swab | No | No | No | AZM ER | Neg | na | na |
| P20 | 22 | Urine | No | Yes | 23S (A2059G) | AZM 1g | na | na | na |
| P21 | 45 | Anorectal swab | No | No | na | AZM ER | Neg | na | na |
| P22 | 30 | Urine | No | Yes | 23S (A2059G), *parC* (G259T) | AZM 1g | Pos | MOX 7d | Neg |
| P23 | 32 | Anorectal swab | No | Yes | na | AZM ER | Neg | na | na |
| P24 | 31 | Anorectal swab | No | No | na | AZM ER | Neg | na | na |
| P25 | 21 | Anorectal swab | No | No | No | AZM ER | na | na | na |
| P26 | 33 | Anorectal swab | No | No | 23S (A2059G) | AZM ER | Pos | MOX 10d | na |
| P27 | 22 | Anorectal swab | No | No | 23S (A2059G), *parC* (G248A) | AZM ER | Pos | MOX 10d | Neg |
| P28 | 51 | Urine | No | No | No | AZM 1g | Neg | na | na |
| P29 ^1^ | 42 | Anorectal swab | No | No | na | AZM ER | Neg | na | na |
| P29 ^2^ | 42 | Anorectal swab | No | No | na | AZM ER | Pos | MOX 10d | Neg |
| P30 | 27 | Anorectal swab | No | No | No | AZM ER | Neg | na | na |
| P31 | 44 | Urine | No | No | na | MOX 10d | Neg | na | na |
| P32 | 49 | Urine | No | na | na | na | na | na | na |
| P33 | 23 | Anorectal swab | No | No | na | AZM ER | Pos | MOX 10d | na |
| P34 | 45 | Urine | No | No | na | AZM ER | na | na | na |
| P35 | 27 | Urine | No | na | na | na | na | na | na |
| P36 | 34 | Anorectal swab | No | Yes | 23S (A2059G) | AZM ER | Pos | na | na |
| P37 | 51 | Urine | No | No | na | AZM ER | na | na | na |
| P38 | 33 | Urine | No | No | *parC* (G259A) | AZM ER | Pos | MOX 10d | Neg |
| P38 | 33 | Anorectal swab | No |  | *parC* (G259A) |  | Pos |  | Neg |
| P39 | 41 | Anorectal swab | No | No | na | AZM ER | Neg | na | na |
| P40 ^1^ | 51 | Anorectal swab | NG, CT | No | na | AZM ER | Neg | na | na |
| P40 ^2^ | 52 | Anorectal swab | No | No | No | AZM ER | Neg | na | na |
| P40 ^3^ | 53 | Anorectal swab | No | No | na | AZM ER | Pos | MOX 10d | Neg |
| P41 | 40 | Anorectal swab | No | No | na | AZM ER | Pos | MOX 10d | Neg |
| P42 | 46 | Anorectal swab | No | No | na | MOX 10d | Neg | na | na |
| P43 | 34 | Urine | No | No | na | AZM ER | Neg | na | na |
| P44 ^1^ | 33 | Anorectal swab | No | No | 23S (A2058T) | AZM ER | Neg | na | na |
| P44 ^2^ | 34 | Anorectal swab | No | Yes | na | MOX 10d | Neg | na | na |
| P45 | 32 | Anorectal swab | No | No | na | AZM ER | na | na | na |
| P46 | 39 | Anorectal swab | NG | No | na | AZM ER | Neg | na | na |
| P47 | 41 | Anorectal swab | No | No | na | AZM ER | Pos | MOX 10d | Neg |
| P48 | 40 | Anorectal swab | No | No | na | AZM ER | Pos | MOX 10d | na |
| P49 | 32 | Anorectal swab | No | No | na | AZM ER | Pos | MOX 10d | na |
| P50 | 27 | Anorectal swab | No | No | na | AZM ER | na | na | na |
| P51 | 33 | Anorectal swab | No | No | na | DOX 7d + MOX 7d | na | na | na |
| P52 | 36 | Anorectal swab | No | No | 23S (A2059G), *parC* (G244A) | AZM ER | Pos | MOX 10d | Neg |
| P53 | 52 | Pharyngeal swab | No | No | No | AZM ER | Neg | na | na |
| P54 | 27 | Anorectal swab | CT | No | na | AZM ER | Neg | na | na |
| P55 | 36 | Urine | No | Yes | na | AZM 1g | Pos | MOX 10d | Neg |
| P56 | 28 | Anorectal swab | No | No | na | AZM ER | Neg | na | na |
| P57 ^1^ | 29 | Anorectal swab | NG | No | 23S (A2058T) | AZM ER | Pos | MOX 10d | Neg |
| P57 ^2^ | 30 | Anorectal swab | No | No | na | MOX 10d | Neg | na | na |
| P58 | 21 | Anorectal swab | No | No | 23S (A2058T) | AZM ER | Pos | MOX 10d | Neg |
| P59 | 31 | Anorectal swab | No | No | na | na | na | na | na |
| P60 | 35 | Urine | No | Yes | 23S (A2058G, A2059G) | AZM 1g | na | na | na |
| P61 | 36 | Anorectal swab | No | No | na | AZM ER | Neg | na | na |
| P62 ^1^ | 45 | Anorectal swab | No | No | 23S (A2058T), *parC* (G259A) | AZM ER | na | na | na |
| P62 ^2^ | 46 | Anorectal swab | CT | No | 23S (A2058T) | AZM ER | Pos | MOX 10d | Neg |
| P63 | 32 | Urine | No | na | na | na | na | na | na |
| P64 | 52 | Urine | No | No | na | na | na | na | na |
| P65 | 34 | Anorectal swab | CT | No | na | AZM ER | Neg | na | na |
| P66 | 54 | Anorectal swab | CT | No | na | MOX 10d | Neg | na | na |
| P67 | 42 | Urine | No | No | No | AZM ER | Neg | na | na |
| P68 | 30 | Urine | No | na | na | na | na | na | na |
| P69 | 42 | Anorectal swab | No | No | na | MOX 10d | Neg | na | na |
| P70 | 24 | Anorectal swab | No | No | na | AZM ER | Pos | MOX 10d | Neg |
| P71 | 38 | Anorectal swab | No | No | na | AZM ER | Neg | na | na |
| P72 | 40 | Anorectal swab | No | No | 23S (A2058G) | AZM ER | Pos | MOX 10d | Neg |
| P73 | 28 | Urine | No | No | na | MOX 10d | Neg | na | na |
| P74 | 36 | Anorectal swab | No | No | na | AZM ER | Pos | AZM ER | na |
| P75 | 45 | Urine | No | No | na | AZM ER | na | na | na |
| P76 | 41 | Anorectal swab | No | Yes | na | AZM ER | Pos | MOX 10d | Neg |
| P77 | 31 | Anorectal swab | No | No | na | AZM ER | Pos | MOX 10d | Neg |
| P78 ^1^ | 31 | Anorectal swab | No | Yes | 23S (A2059G) | AZM ER | Pos | MOX 10d | Neg |
| P78 ^2^ | 32 | Anorectal swab | No | No | na | AZM ER | na | na | na |
| P79 | 46 | Urine | No | Yes | na | AZM ER | Neg | na | na |
| P80 | 24 | Anorectal swab | CT | Yes | na | AZM ER | Pos | MOX 10d | Neg |
| P81 | na | Urine | No | na | na | na | na | na | na |
| P82 | na | Urine | No | na | na | na | na | na | na |
| P83 | 39 | Anorectal swab | No | No | 23S (A2059G) | AZM ER | na | na | na |
| P84 | na | Anorectal swab | No | No | 23S (A2059G) | AZM ER | na | na | na |
| P85 | na | Anorectal swab | NG | No | No | AZM ER | na | na | na |
| P86 | 57 | Anorectal swab | CT | No | No | AZM ER | Neg | na | na |
| P87 | 31 | Anorectal swab | No | No | 23S (A2059G) | AZM ER | na | na | na |
| P88 | 71 | Urine | No | No | No | AZM 1g | na | na | na |
| P89 | 58 | Urine | No | No | 23S (A2059G) | AZM 1g | na | na | na |
| P90 | 26 | Anorectal swab | No | No | 23S (A2059G) | AZM ER | na | na | na |
| P91 | 47 | Anorectal swab | No | No | 23S (A2059G) | AZM ER | na | na | na |
| P92 | na | Anorectal swab | CT | Yes | na | AZM ER | Neg | na | na |
| P93 | 55 | Anorectal swab | No | No | na | AZM ER | na | na | na |
| P94 | na | Urine | No | No | 23S (A2059G), *gyrA* (C270T) | AZM ER | na | na | na |
| P95 | 45 | Anorectal swab | No | No | 23S (A2059G), *gyrA* (G285C) | AZM ER | na | na | na |
| P96 | na | Anorectal swab | No | No | na | AZM ER | na | na | na |

^1,2,3^ = First, second, third infection

CT = *C. trachomatis*

NG = *N. gonorrhoeae*

AZM = azithromycin

ER = extended regiment

MOX = moxifloxacin

DOX = doxycycline

na = not available
